# Improvement of the initial management of sarcomas after the dissemination of evidence-based guidelines depends on the primary sarcoma location: a population-based study

**DOI:** 10.1186/s12885-015-1225-x

**Published:** 2015-04-02

**Authors:** Karine Ligier, Carlos Maynou, Xavier Leroy, Yves-Marie Robin, Philippe Martin, Stéphanie Clisant, Florence Richard, Nicolas Penel

**Affiliations:** 1 Registre général des cancers de Lille et de sa région, GCS C2RC, CHRU de Lille, Hôpital Calmette - Pavillon Breton, Boulevard du Professeur Jules Leclercq, 59037 Lille, Cedex France; 2 SIRIC ONcoLille, Plateforme 4 (Méthodologie et Recherche Clinique), Lille, France; 3 Département d’Orthopédie A, Hopital Roger Salengro, CHRU de Lille, Lille, France; 4 Département de Pathologie, CHRU de Lille, Lille, France; 5 Département de Pathologie, Centre Oscar Lambret, Lille, France; 6 Service de Cancérologie, Clinique du Bois, Lille, France; 7 Unité de Recherche Clinique, Centre Oscar Lambret, Lille, France; 8 Institut Pasteur Lille, INSERM U744, Lille, France; 9 Département de Cancérologie Générale, Centre Oscar Lambret, 3, rue F Combemale, 59020 Lille, Cedex France

**Keywords:** Sarcoma, Guidelines, Compliance, Multidisciplinary committee, Quality of care, Registry

## Abstract

**Background:**

Improvement of the initial management of sarcomas after the dissemination of evidence-based guidelines depends on the primary sarcoma location: a population-based study.

To improve the initial management of adult sarcomas, a regional expert team in Northern France performed two actions: dissemination of evidence-based guidelines (EBG) for the management of soft tissue/visceral sarcoma and yearly educational symposia. The aim of this study was to measure the impact of the dissemination of EBG on the key-indicators of adult sarcoma management.

**Methods:**

We conducted a before-after population-based study (before: 2005 with 63 cases, after: 2008–2009 with 86 cases) in the Lille area (Northern France urban/sub-urban area with 800,000 inhabitants). The following were the key-indicators of adult sarcoma management: pre-therapeutic biopsy, appropriate tumour and chest imaging, expert interdisciplinary discussion, expert interdisciplinary discussion before the first treatment and in operated cases, the rate of R0 resection.

**Results:**

There was no statistically significant difference in patient and tumour characteristics for the two time periods in terms of gender, prior cancer, primary location, histological subtype, grade, size, metastasis and lymph node involvement. There was no statistically significant improvement in primary tumour imaging (83 versus 87%), chest imaging (67 vs 71%), pre-therapeutic biopsy (57 vs 58%). There was an improvement in expert multidisciplinary discussion (37 vs 45%) or discussion before the first treatment (26 vs 44%) but no statistically significant. However, when soft tissue and bone sarcomas were analysed separately, we observed statistically significant improvements in expert multidisciplinary discussion (50 vs 74%, p = 0.02) and R0 resection rate (58 vs 91%, p = 0.002). In contrast, in cases of visceral sarcoma, there was no improvement in expert multidisciplinary discussion (10 vs 16%, p = 0.7) or in R0 resection (88 vs 81%, p = 0.7).

**Conclusions:**

The dissemination of EBG was associated with a limited improvement in sarcoma management when measured in this before-after population-based study, and this improvement was dependent on the primary location of the tumour. Efforts to implement these guidelines by all surgical teams that could treat sarcoma, including visceral sarcoma, need to be made.

## Background

Sarcomas account for approximately 2% of all adult cancers. Sarcomas are heterogeneous in terms of their histological sub-types, histological grades, locations (soft tissue, bone and viscera), clinical behaviours and prognosis. Nevertheless, at the early stages of tumour development, the optimal management of these rare cancers is based on a few key-rules: standardised radiological check-ups, biopsies of any deep and large soft tissue masses and bone tumours and tailored treatment that is best defined after a case by case analysis by a multidisciplinary committee, including a surgeon, radiotherapist, medical oncologist, pathologist and radiologist, with expertise in sarcoma management. The primary goal of the management of non-metastatic sarcoma is to achieve a large en-bloc resection. The achievement of clear margins resection (R0) remains the key-prognostic factor for local control [1-3].

In France, recent efforts have been made to improve the management of rare cancers, including sarcoma. Since 1995, evidence-based guidelines for the management of sarcoma (“*Standard-Options-Recommendations*” for the management of soft tissue and visceral sarcoma) have been widely disseminated to health professionals. The sarcoma guidelines were updated in 2006, and a second version of them was widely disseminated with the help of regional cancer networks [4]. Later (2010), the French Cancer Institute (INCa, *“National du Cancer”*) labelled some regional and inter-regional experts as centres for organising expert inter-disciplinary committees (EIC) dedicated to sarcoma management.

Since 1999, physicians from the Centre Oscar Lambret (comprehensive cancer centre) and from the Lille University Hospital have organised weekly EIC dedicated to sarcoma management. Since 2006, these physicians have also organised yearly educational symposia to support the dissemination of evidence-based clinical practice guidelines and improve the quality of sarcoma management. Furthermore, guidelines have been posted on the Web-site of the Regional Reference Cancer Center; the link to these web-accessible guidelines have been copy-pasted on all multidisciplinary committee reports sent to general practitioners and treating physicians.

The purpose of the current study was to measure the impact of the dissemination of the clinical practice guidelines using a before-after analysis measuring some key-indicators in a general population of the Lille area.

## Methods

### Study population

This before-after population-based study included all patients over 18 years of age with a diagnosis of sarcoma in 2005 and 2008–2009. These patients were selected from the general cancer registry of the Lille area [5]. The Lille area is an urban and sub-urban area located in northern France with approximately 800,000 inhabitants. The sarcoma morphologies that were included in the present study were as follows (coded with International Classification of Diseases for Oncology - third edition - ICD-O 3): 8710/3, 8711/3, 8800/3-8933/3, 8935/3-8940/3, 8950/3-8951/3, 8963/3-8964/3, 8980/3-8991/3, 9040/3-9044/3, 9120/3-9260/3, 9290/3, 9330/3,9342/3, 9364/3, 9365/3, 9473/3, 9480/3, 9508/3 and 9539/3-9581/3.

### Data

The data were collected from medical records and included civil status, date of diagnosis, topography and morphology of the cancer coded by ICD-O 3, the staging performed, the date of the EIC dedicated to the sarcoma, the characteristics of the tumour and the treatment (date, type and location).

We defined “expert teams” as those that were (a) trained in interdisciplinary sarcoma management and (b) organised weekly interdisciplinary committees dedicated to sarcoma management. Later (2010), these teams were labelled by the French Cancer Institute (INCa) for the management of sarcoma patients.

### Key performance indicators

We collected data regarding the key-indicators of the initial examination and diagnosis of sarcoma [6] to evaluate the compliance of expert teams to the procedures described in the EBG for the initial check-up. This included biopsy before planned curative-intent surgery, appropriate primary tumour imaging and appropriate chest imaging. Some key-indicators related to the management process were also collected: rate of incident cases discussed by an EIC dedicated to sarcoma, rate of incident cases discussed within the three months following the diagnosis, the rate of incident cases discussed before the first treatment (pre-biopsy EIC) and the rate of R0 resection among operated patients [7].

### Statistical analyses

The descriptive analysis used median or mean and extreme values for continuous variables and frequency for categorical variables. The comparisons were performed for two periods: 2005 (before the dissemination of the clinical practice guidelines) and 2008–2009 (after the dissemination). The Pearson’s Chi-2 test and Fischer’s exact test were used as appropriate. We hypothesised that the management of sarcoma was significantly different depending on the primary location of the sarcoma, soft tissue and bone sarcoma versus visceral sarcoma. Both location categories were analysed separately. Furthermore, we have explored the factors associated with R0 resection achievement. The level of significance was set at 0.05.

The analyses were conducted using StataIC 11 software (ref: StataCorp. Stata: Statistical Software Release 11 College Station. TX. StataCorp LP).

### Regulations and ethics

This registry was authorised by the National Ethical Committee (“Commission Nationale Informatique et Libertés”, CNIL).

## Results

### Patient and tumour characteristics

In the Lille area, 63 and 86 adult sarcomas were diagnosed in 2005 and 2008–2009, respectively. The management of sarcoma patients (surgery, radiotherapy and chemotherapy) have been done in all hospitals covered by the Regional Registry. However, the French National Cancer Institute strongly supports the management of these patients in 2 reference centers: Lille University Hospital and Oscar Lambret Cancer Center (the 2 expert teams).” The tumour and patient characteristics are summarised in Table 1. The median age of the patients was 58.9 years (18.5-92.6). There were 51 men (34.2%) and 98 women (65.8%). The main common histological subtypes were: liposarcoma (21, 14.1%), leiomyosarcoma (17, 11.4%), dermatofibrosarcoma (9, 6.0%), synovial sarcoma (9, 6.0%), chondrosarcoma (8, 5.4%) and gastro-intestinal stromal tumour (8, 5.4%). Thirty-three patients (22.2%) had a previous history of cancer (mainly breast cancer, 17 cases). The mean tumour size was 7.8 cm (1.0-25.0) in 2005 and 9.8 cm (1.4-35.0) in 2008–2009. Seventy-four percent of the tumours were profound. There was no difference between the two periods in any of the characteristics analysed (Table 1).

**Table 1:** **Tumour and patient characteristics**

Characteristics		2005		2008-2009		p univariate analysis
		*n.*	(%)	*n.*	(%)	
Cases		63	-	86	-	
Gender	Men	21	33.3	30	34.9	0.844
	Women	42	66.7	56	65.1	
Age at diagnosis	<60 years	34	54.0	46	53.5	0.954
	≥60 years	29	46.0	40	46.5	
Prior cancer	Yes	16	25.4	17	19.8	0.414
	No	47	74.6	69	80.23	
Primary location	Soft tissue	34	54.0	37	43.0	0.145
	Bone	8	12.7	6	7.0	
	Gynaecological viscera	10	15.9	15	17.4	
	Other viscera	11	17.4	28	32.6	
Histological subtypes	Dermatofibrosarcoma	4	6.3	5	5.8	0.412
	Kaposi sarcoma	0	0.0	4	4.7	
	Leiomyosarcoma	8	12.7	9	10.5	
	Liposarcoma	12	19.0	9	10.5	
	Synovial sarcoma	1	1.6	8	9.3	
	Other soft tissue sarcomas	27	42.9	39	45.3	
	Ewing sarcoma	2	3.2	1	1.2	
	Chondrosarcoma	4	6.3	4	4.7	
	Osteosarcoma	1	1.6	1	1.2	
	Other osseous sarcomas	1	1.6	1	1.2	
	Gastro-intestinal stromal Tumour	3	4.8	5	5.8	
Histological grade	Low grade	15	23.8	27	31.4	0.386
	Intermediate	12	19.0	8	9.3	
	High	22	34.9	26	30.2	
	Not applicable	4	6.4	8	9.3	
	Unknown	10	15.9	17	19.8	
Location	Superficial	18	28.6	19	22.1	0.553
	Deep	45	71.4	66	76.7	
	Unknown	0	0	1	1.2	
Size	<5 cm	19	30.2	16	18.6	0.243
	> = 5 cm	30	47.6	50	58.1	
	Unknown	14	22.2	20	23.3	
Lymph node involvement	Yes	6	9.5	6	7.0	0.752
	No	53	84.1	72	83.7	
	Unknown	4	6.4	8	9.3	
Metastasis at diagnosis	Yes	17	27.0	16	18.6	0.383
	No	41	65.1	65	75.6	
	Unknown	5	7.9	5	5.8	

### Key performance indicators: before-after analysis

When comparing the data from 2005 and 2008–2009, we observed, among cases discussed in sarcoma management, a statistically significant improvement in the proportion of cases that were discussed within ninety days of diagnosis (78.2 vs 97.4%, p = 0.023) and, among cases that were operated, an improvement in the proportion of successful R0 resections (67.3 vs 86.4%, p = 0.024). We observed also an improvement in the rate of cases discussed by an EIC (36.5 vs 45.3%) and rate of cases discussed by an EIC before the first treatment (26.0 vs 44.4%) but these differences were not statistically significant. There was no difference in the rate of accurate primary tumour imaging, rate of accurate chest imaging, rate of cases with biopsy (Table 2).

**Table 2:** **Changes in key-indicators**

Characteristics		2005		2008-2009		p univariate analysis
		*n.*	(%)	*n.*	(%)	
Cases		63	-	86	-	
Primary tumour imaging	Yes	52	82.5	75	87.2	0.427
	No and unknown	11	17.5	11	12.8	
Chest CT scan	Yes	42	66.7	61	70.9	0.578
	No and unknown	21	33.3	25	29.1	
Biopsy	Yes	36	57.1	50	58.1	0.903
	No	27	42.9	36	41.9	
Case discussed in expert interdisciplinary committee	Yes	23	36.5	39	45.3	0.279
	No	40	63.5	47	54.7	
Case discussed within the 90 days following the diagnosis (1)	Yes	18	78.2	38	97.4	0.023
	No	5	21.8	1	2.6	
Case discussed before the first treatment (2)	Yes	6	26.0	16	44.4	0.155
	No	17	74.0	20	55.6	
First surgery by expert team (3)	Yes	25	45.5	32	48.5	0.740
	No	30	54.5	34	51.5	
Resection (3)	R0	37	67.3	57	86.4	0.024
	R1 or R2	16	29.1	8	12.1	
	Unknown	2	3.6	1	1.5	

(1) – Among cases discussed in multidisciplinary meeting.

(2) – Among cases discussed in multidisciplinary meeting and treated.

(3) – Among operated patients.

### Before-after analysis according to primary location

Improvements in the management of adult sarcoma were observed for soft tissue and bone sarcomas (Table 3): we observed a statistically significant increase in the proportion of EIC discussions (50.0 vs 74.4%, p = 0.020), EIC discussions within 90 days after diagnosis (76.2 vs 100%, p = 0.007) and R0 resections (57.9 vs 91.4%, p = 0.002). An improvement was noted in the proportion of accurate primary tumour imaging (83.3 vs 95.4%), EIC discussion before first treatment (23.8 vs 46.7%) and first surgery by an expert team (42.1 vs 62.9%) but the improvement was not statistically significant (p = 0.089, p = 0.097 and p = 0.076, respectively). Among large (>3 cm) or deep soft tissue tumours, the proportion of pre-therapeutic biopsy did not show a statistically significant increase (42.3% and 55.9% for 2005 and 2008–2009, respectively, p = 0.297). There was no statistically significant improvement in patients with visceral sarcoma, for which only 16.3% were discussed by an EIC.

**Table 3:** **Changes in key-indicators according to primary location**

Indicator	2005		2008-2009		p univariate analysis
	n.	%	n.	%	
*Soft tissue and bone sarcoma*					
Cases	42	-	43	-	
Accurate primary tumour imaging	35	83.3	41	95.4	0.089
Chest CT scan	30	71.4	33	76.7	0.576
Biopsy	21	50.0	16	60.5	0.332
Expert inter-disciplinary committee discussion	21	50.0	32	74.4	0.020
Discussion within the 90 days following the diagnosis (1)	16	76.2	32	100.0	0.007
Discussion before first treatment (2)	5	23.8	14	46.7	0.097
First surgery by expert team (3)	16	42.1	22	62.9	0.076
R0 Resection (3)	22	57.9	32	91.4	0.002
*Viscera sarcoma*					
Cases	21	-	43	-	
Accurate primary tumour imaging	17	81.0	34	79.1	1.000
Chest CT scan	12	57.1	28	65.1	0.536
Biopsy	15	71.4	24	55.8	0.229
Expert inter-disciplinary committee discussion	2	9.5	7	16.3	0.706
Discussion within the 90 days following the diagnosis (1)	2	100	6	85.7	1.000
Discussion before first treatment (2)	1	50.0	2	33.3	1.000
First surgery by expert team (3)	9	52.9	10	32.3	0.161
R0 Resection (3)	15	88.2	25	80.7	0.694

(1) – Among cases discussed in multidisciplinary meeting.

(2) – Among cases discussed in multidisciplinary meeting and treated.

(3) – Among operated patients.

### Ro resection, discussion in multidisciplinary meeting

The achievement of R0 resection was associated with EIC discussion in cases of bone and soft tissue sarcoma (0.018) (Table 4). The achievement of R0 resection in first surgery was associated with the surgery being performed by an expert team (0.045), especially for soft tissue and bone sarcomas (0.027).

**Table 4:** **Factors associated with the achievement of R0 resection: univariate analysis**

	R0 Resection n (%)	R1, R2, not done n(%)	p univariate analysis
Discussion in expert inter-disciplinary committee			
Yes	41 (82.0)	9 (18.0)	
No	53 (74.7)	18 (25.3)	0.339
Discussion in expert inter-disciplinary committee: bone and soft tissue sarcomas			
Yes	39 (83.0)	8 (17.0)	
No	15 (57.7)	11 (42.7)	0.018
Discussion in expert inter-disciplinary committee: visceral sarcomas			
Yes	2 (66.7)	1 (33.3)	
No	38 (84.4)	7 (15.6)	0.429
First surgery by expert team			
Yes	42 (73.7)	15 (26.3)	
No	36 (56.3)	28 (43.8)	0.045
First surgery by expert team: bone and soft tissue sarcomas			
Yes	28 (73.7)	10 (26.3)	
No	17 (48.6)	18 (51.4)	0.027
First surgery by expert team: visceral sarcomas			
Yes	14 (73.7)	5 (26.3)	
No	19 (65.5)	10 (34.5)	0.751

Percentage by row.

## Discussion

The key-findings of this study are as follows: (a) the dissemination of evidence-based guidelines was associated with an improvement in sarcoma management in this before-after population-based study and (b) the improvement of sarcoma management depends on the primary location of the tumour, with an obvious improvement in cases of soft tissue and bone sarcomas.

### Improvement of management

The role of the dissemination of evidence-based guidelines in the improvement of sarcoma management has been explored in different studies, and the results of these studies have been contradictory. In the 1990s, Ray-Coquard *et al.* conducted several before-after studies in the Rhone-Alpes region regarding breast and colo-rectal cancer management and found that the dissemination of guidelines was not associated with a statistically significant improvement in the compliance to those guidelines [8,9]. In another before-after population-based study conducted in the Netherlands (1998–1999 versus 2006; 79 versus 40 cases), Jansen-Landhee *et al.* showed a statistically significant improvement in terms of compliance to radiological check-up, pre-treatment biopsy recommendation and second opinion histological diagnosis [10]. In our experience, we have found that the dissemination of guidelines alone was associated with a partial improvement in the compliance to guidelines. Nevertheless, we have measured a statistically significant improvement in the proportion of cases discussed by an EIC in the 90 days following diagnosis (78 versus 97%, p = 0.023) and an improvement in the proportion of successful R0 resections (67 versus 86%, p = 0.024).

The most interesting finding of the present study was that the improvement of sarcoma management depends on the primary tumour location. The improvement was obvious in cases of soft tissue and bone sarcoma. This can be easily explained as follows: most sarcomas are located in the limbs and trunk, and most sarcoma patients are referred to oncology surgical teams or orthopaedic teams that are very involved in the organisation of the weekly expert inter-disciplinary committee. In contrast, patients with visceral sarcomas are referred to a variety of surgical teams (digestive, gynaecological, head and neck, neurological, etc.) that treat patients according to their own guidelines, which can be different from the sarcoma guidelines. Moreover, the differential diagnosis of visceral carcinoma and visceral sarcoma remains difficult before obtaining pathological data. There is an important difference between soft tissue masses and visceral masses; most soft tissue masses are sarcomas whereas most visceral masses are carcinomas. Therefore, soft tissue masses are managed *a priori* as sarcoma whilst visceral sarcomas are first treated as carcinoma, with no systematic pre-surgical biopsy. Because sarcoma could arise in any part of the body, efforts have to be made to disseminate guidelines to all surgical teams and to (re)-explain the importance of EIC before any surgical treatment, regardless of the primary site of the tumour.

### Actual management

Ray-Coquard *et al.* showed in a retrospective analysis of 100 sarcoma medical charts that the factors associated with conformity to guidelines were as follows: discussion by an expert interdisciplinary committee before surgery, treatment by an expert team and management with an expert network [6]. Furthermore, Sampo et al. showed that the local control rate has been higher when pre-treatment biopsy is performed and when patients are operated on in high-volume centres [11]. We found that, despite the dissemination of evidence-based guidelines, the management of sarcoma remains suboptimal, especially in non-expert centres. Improvement in compliance to guidelines must occur for chest imaging, biopsy before treatment and EIC discussion, especially before treatment. Our study shows that 77% of patients with soft tissue and bone sarcoma underwent chest imaging, and 56% of patients with deep or large tumours had a biopsy; this finding is consistent with the data in the literature. For example, Heudel et al. demonstrated, in a non-exhaustive retrospective study with more than 600 patients, that only 64% of patients underwent accurate chest imaging and only 22% of patients with a soft tissue mass measuring more than 3 cm underwent a pre-treatment biopsy [12]. In our study, R0 resection was more frequent for bone and soft tissue sarcomas when surgery was conducted by an expert team. Several studies have stressed that there is a statistically significant difference in terms of compliance to guidelines between expert (or university) teams and non-expert (or non-university) teams [7,11,13-18].

### Strengths and limitations

The main strengths of the present study is the population-based design and the exhaustiveness of the recruitment based on registry [5]. Furthermore, the collection of data, based on medical files, was precise. Our study has also some limitations. Regarding the limited number of cases, the statistical tests have to be interpreted with caution: when the magnitude of difference of proportions between the two period studied suggests an improvement, the non-significance do not necessarily mean the absence of effect but reflect a lack of power. We have analysed the factors associated with achievement of R0 resection. Nevertheless, regarding the limited number of cases, we were not able to conduct a multivariate analysis.

Furthermore, this was a before-after retrospective study with some inherent imprecision, we cannot distinguish the changes due to the intervention from the simple time trend or other activities (e.g. national of international interventions) that can influence the outcome: a direct causality between the analysed parameters and outcomes cannot be shown, especially for the causality between the improvement of medical management and the dissemination of the guidelines. At the end, statistically significant association does not mean correlation; we cannot formally attribute the observed changes to the diffusion of guidelines. However, our study showed significant improvements of some key-indicators describying the management of limb and chest or abdominal wall sarcoma. On the contrary, there was no improvement of visceral sarcoma, not covered by the evidence-based guidelines. So we may assume that this is partly caused by the dissemination of the clinical practice guidelines.

## Conclusions

This study showed that the dissemination of guidelines could be associated with improvement in sarcoma management. Efforts to implement these guidelines by all surgical teams that could treat sarcoma, including visceral sarcoma, are necessary.
